# The bounds of meta-analytics and an alternative method

**DOI:** 10.4178/epih.e2024016

**Published:** 2024-01-07

**Authors:** Ramalingam Shanmugam, Mohammad Tabatabai, Derek Wilus, Karan P. Singh

**Affiliations:** 1 School of Health Administration, Texas State University, San Marcos, TX, USA; 2 Meharry Medical College, School of Graduate Studies, Nashville, TN, USA; 3 Department of Epidemiology and Biostatistics, School of Medicine, The University of Texas at Tyler, Tyler, TX, USA

**Keywords:** Heterogeneity, Risk, Cognitive impairment, COVID-19, Vaccination

## Abstract

**OBJECTIVES:**

Meta-analysis is a statistical appraisal of the data analytic implications of published articles (*Y*), estimating parameters including the odds ratio and relative risk. This information is helpful for evaluating the significance of the findings. The Higgins I^2^ index is often used to measure heterogeneity among studies. The objectives of this article are to amend the Higgins I^2^ index score in a novel and innovative way and to make it more useful in practice.

**METHODS:**

Heterogeneity among study populations can be affected by many sources, including the sample size and study design. They influence the Cochran Q score and, thus, the Higgins I^2^ score. In this regard, the I^2^ score is not an absolute indicator of heterogeneity. Q changes by bound as *Y* increases unboundedly. An innovative methodology is devised to show the conditional and unconditional probability structures.

**RESULTS:**

Various properties are derived, including showing that a zero correlation between Q and *Y* does not necessarily mean that they are independent. A new alternative statistic, S^2^, is derived and applied to mild cognitive impairment and coronavirus disease 2019 vaccination for meta-analysis.

**CONCLUSIONS:**

A hidden shortcoming of the Higgins I^2^ index is overcome in this article by amending the Higgins I^2^ score. The usefulness of the proposed methodology is illustrated using 2 examples. The findings have potential health policy implications.

## GRAPHICAL ABSTRACT

[Fig f1-epih-46-e2024016]

## Key Message

An approach to overcome the hidden shortcomings of Higgens I^2^ in meta-analysis. The approach has potential health policy implications.

## INTRODUCTION

The genesis of meta-analysis can be traced to the work of an eminent statistician [1] who compared evidence from several studies on typhoid inoculation. Meta-analysis is intended to identify patterns of similarities and differences among studies with the same aim. Glass et al. [2] and others have discussed it in detail. Meta-analysis has been criticized for averaging the differences of studies with sample data from heterogeneous populations. A systematic review precedes the meta-analysis for the sake of appraising critical evidence in the publications. A meta-analysis is performed in sequential steps. As exemplified by research aiming to establish the impact of vitamin D on protecting patients from coronavirus disease 2019 (COVID-19) [3-23], the steps include focusing on establishing research questions, formulating the population, conducting a literature search for published results, selecting published studies of appropriate quality, and evaluating whether the summary measures in studies are comparable, whether the model to integrate the studies should involve fixed or random effects, and whether the heterogeneity among the study populations is acceptable in order for the findings of the meta-analysis to yield meaningful insights into the issue at hand.

Other noteworthy recent meta-analytic studies include Pearson [24] and more [25-31]. Recently, Hong et al. [14] published an article on the importance of meta-analysis in the journal of the Korean Society of Epidemiology, *Epidemiology and Health*. Using the inverse of the estimated variance of the studies, the fixed type of meta-analysis provides a weighted average estimate. When populations are heterogeneous, the random type is appropriate, and it is applied with inverse variance as weights or no weights at all. A disadvantage of meta-analysis is that the sources of bias are not accounted for in the calculations of heterogeneity. When the findings of studies lack significance, the results are often not reported in any publication; this phenomenon is known as publication bias (or the “file drawer” problem). The role of publication bias is beyond the scope of this paper. The reader is referred to Borenstein et al. [8] for the role of the Higgins score in relation to the heterogeneity of the sampled populations in meta-analyses and to Chernikova et al. [32] and Blumenfeld [33] for a discussion of simulation-based learning meta-analysis. The probability distribution of the Higgins statistic, I^2^ ≥0, is assumed to have a chi-squared distribution. However, in some studies, when *Q≥df* is not true, I^2^ does not have a chi-squared distribution. Note that *Q* and *df* refer to the Cochran Q score and degrees of freedom (df), respectively. In this manuscript, a modified approach is given to rectify this shortcoming in the Higgins statistic-based approach. The approach is illustrated by applying it to 2 examples—cognitive impairment and COVID-19 vaccination—for meta-analysis.

## MATERIALS AND METHODS

### An alternative meta analytic approach

Epidemiologists, biostatisticians, and investigators in other disciplines utilize the Higgins statistic, $I^{2}=\frac{\left(Q-\mathrm{df}\right)}{O}$, in meta-analysis, where Q and df are Cochran’s score and df, respectively. It is straightforward and obvious that I^2^ ≥0 and it is necessary that *Q≥df* while conducting a meta-analysis. In some studies, this requirement is not satisfied. Consequently, the Higgins statistic, I^2^, does not have a chi-squared distribution. We offer a modified approach to rectify this shortcoming in the Higgins statistic-based approach as follows:

The probability pattern of the Higgins statistic $I^{2}=\frac{\left(Q-\mathrm{df}\right)}{O}$ is explored, and the expression *Corr(Q,y)*= 0 is utilized (Supplementary Material 1), where Q and df are the Cochran Q score and df, respectively. The random number, *Y=y*, corresponding to the number of studies on a topic available for the meta-analyst to consider at a point in time, must be *y*= 2,3,...,*θ*, in a meta-analysis, where *θ* is an unknown upper-bound uniform parameter. Its transformation $u=\frac{y-1}{\theta }$ follows a probability density function (pdf) $f\left(u|\theta \right)={\left(1-\frac{1}{\theta }\right)}^{-1}$. An additional transformation *w*=-2ln*u* has the sample space $0<w<-2\ln\left(\frac{1}{\theta }\right)$, and its pdf is $f\left(w|\theta \right)={\left(1-\frac{1}{\theta }\right)}^{-1}\left(\frac{1}{2}\right)e^{-\frac{w}{2}}$. We note that $E\left(w|\theta \right)\approx \left(1-\frac{1}{\theta }\right)$ and the variance is $Var\left(w|\theta \right)\approx 3{\left\{E\left(w|\theta \right)\right\}}^{2}$ (Supplementary Material 2). The survival function of *w* is $Pr\left[W>m\right]=1-{\left(1-\frac{1}{\theta }\right)}^{-1}\left(1-e^{-m/2}\right);m\geq 2$. The incremental rate of researchers performing additional studies is $h\left[w|m\right]=\frac{f\left(w|\theta \right)}{Pr\left[W>m\right]}=\frac{e^{-\left(\frac{w}{2}\right)}}{2\left[\left\{1-\frac{1}{\theta }\right\}-\left\{1-e^{-\left(\frac{w}{2}\right)}\right\}\right]}$, which stabilizes at the asymptote ${\left[1+e^{\left(\frac{m}{2}\right)}\left(1-\frac{2}{\theta }\right)\right]}^{-1}$. The conditional pdf of the statistic *Q* is, for a given *w* (Supplementary Material 2).


(1) $$f\left(Q|w\right)=e^{-\left(\frac{Q}{2}\right)}Q^{\left(\frac{\left\{\theta e^{-\frac{w}{2}}-1\right\}}{2}\right)-1}/2^{\left(\frac{\left\{\theta e^{-\frac{w}{2}}-1\right\}}{2}\right)}\Gamma \left(\frac{\left\{\theta e^{-\frac{w}{2}}-1\right\}}{2}\right);Q>0;0<w<2\ln\theta ;\theta \geq 1$$

Consequently, $E\left(Q\right)=\left(\frac{\theta -1}{2}\right)$ and $Var\left(Q\right)=\frac{\left(\theta +11\right)\left(\theta -1\right)}{12}$. We have shown that *Cov(df,Q)*=0 and *Corr(df,Q)*=0 (Supplementary Material 3). We have obtained a statistical procedure to find the critical value of the new statistic,


(4) $$S^{2}={\left[\frac{I^{2}-E\left(I^{2}\right)}{\sqrt{Var\left(I^{2}\right)}}\right]}^{2}=\chi_{1df}^{2}={\left[\frac{\left\{\frac{\left(\theta +11\right)}{6\left(\theta -1\right)}-\left(1+\frac{df}{Q}\right)\right\}}{\sqrt{3\left|1-\frac{\left(\theta +1\right)}{2\left(\theta -1\right)}\right|}}\right]}^{2}$$

as the expression (4) follows a chi-squared distribution with 1 df. In other words, the p-value of a data base S^2^ is $\mathrm{Pr}\left(S^{2}\geq \chi_{1,p-value}^{2}\right)=p-value$. These results would help the practitioner to have more confidence in conducting meta-analysis.

### Ethics statement

In the article, 2 publicly available data sets were used for illustrating the usefulness of the proposed methodology. Informed consent was not required.

## RESULTS

### Example 1

As an illustration, we consider the recent data collected by Chen et al. [34] and Chen et al. [35] on the global prevalence of mild cognitive impairment (MCI) among elder adults living in nursing homes. The occurrence of MCI is caused by aging and/or dementia. The data they analyzed in various studies using the statistical software Stata, compiling the Q-values and the df from 53 published articles in 17 countries, are reproduced in Table 1. They concluded that there is significant heterogeneity in the studies. The Higgins statistic, I^2^, has been described to follow the chi-squared distribution, whose sample space should be non-negative (that is, I^2^ ≥0). The values of are negative (Table 1, last column) in the data for Europe and Central Asia and for the upper middle-income category. The negative values of I^2^ clearly attest that the Higgins statistic does not always follow the chi-squared distribution. Hence, a refined version of the Higgins statistic is a necessity, and such a revised version is our statistic, S2, whose values are displayed in Table 1.

### Example 2

Parents were concerned about vaccinating their children with the then-untested COVID-19 vaccine. A combined worldwide study using a meta-analysis was used to probe patterns in these concerns. A total of 98 papers across 69 different countries with 413,590 participants were examined by Alimoradi et al. [4]. The authors found that countries’ income level, location, and data collection methods were significant moderators of parents’ willingness to vaccinate their children against COVID-19. The data collection method was another significant factor influencing parental willingness. Studies collected using phone interviews had the lowest prevalence of willingness. None of the studies were thought to have exhibited heterogeneity.

Once again, the Higgins statistic, I^2^, exhibited negative values which violate the required non-negative sample space of the chi-squared distribution (see the last column in Table 2) in the data for all groupings. A refined version of the Higgins statistic is, once again, a necessity. For comparison, our revised statistic, I^2^, is displayed in Table 2.

## DISCUSSION

A word of caution is necessary when interpreting the Higgins I^2^ value and its impact. There are 3 challenges in using the Higgins score, I^2^: (1) It is mentioned by Higgins et al. [36] that I^2^ is the percentage of variation across the studies that is due to heterogeneity rather than sheer chance. Khan [16] commented that “…. The I^2^ values of 25%, 50%, and 75% indicate low, moderate, and high heterogeneity, respectively, among the population effect sizes. I^2^ ≤ 25% of studies are considered to be homogeneous.” (2) *Corr (Q,Y)*= 0 does not imply that *Q* and *Y* are independent. (3) The I^2^ statistic can be negative when Q is less than df. For this situation, it is commented by Higgins et al. [36]: “Negative values of the I^2^ are put equal to zero so that I^2^ is between 0% and 100%.” This causes users to doubt the validity of the score and have less confidence in using it. These shortcomings are overcome by our refinement of the Higgins score, which we explain below:

The exact probability structure of the popularly utilized Higgins score in meta-analytic studies to assess the consistency of the findings in various studies about a healthcare topic is derived. With this probability structure, a method of finding the p-value for the Higgins score and its interpretation is devised and demonstrated. The exact new expression (4) for the score S2 is a refined version of the Higgins standardized score, which follows the chi-squared distribution with 1 df. With these new innovative results, meta-analytic researchers do not have to follow the subjective interpretations of the estimated Higgins score. Instead, the researchers could obtain the p-value for the calculated standardized S2 score based on the chi-squared distribution and conduct an objective, exact interpretation. The values of the new score S^2^ are objective. The authors show both the conditional and unconditional probability structures of the Higgins statistic, including how the correlation between *Q* and *Y* is derived and utilized for *Q* and *Y* to be uncorrelated and independent.

Had Higgins followed the line of the traditional thinking of statistical discipline, he could have defined the I^2^ score as the ratio $\frac{Q}{\left(Q-df\right)}$, and a larger value would have demonstrated heterogeneity. On the contrary, following Higgins’ definition, we ought to interpret that a smaller p-value for the chi-squared distribution implies heterogeneity. It is a tradition in the statistical discipline that a larger value of a statistic refers to significance. One can do objectively better than what is known to date regarding the implications of the Higgins score.

In conclusion, the Higgins I^2^ score stochastically follows a chi-squared distribution with (*n*-1) df, where *n* is the number of studies considered. In all applications, if the score is less than the df, the difference is simply considered to be 0. This zone of the probability area might not be negligible. The authors overcame this hidden shortcoming of the Higgins I^2^ statistic by amending it. The usefulness of the proposed methodology is illustrated by using 2 examples: (1) the global prevalence of MCI among elder adults living in nursing homes, and (2) data on vaccinating children by then-untested COVID-19 vaccines. The findings of this article have potential health policy implications.

## Figures and Tables

**Figure f1-epih-46-e2024016:**
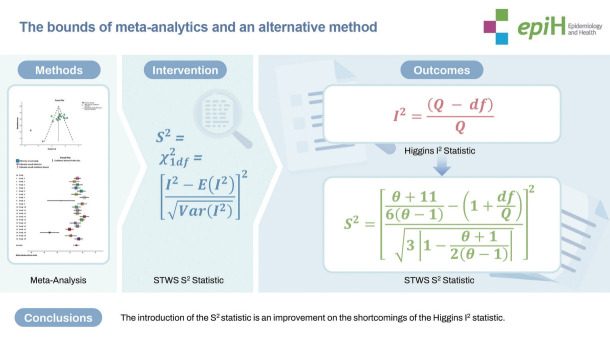


**Table 1.** Results of a meta-analysis on the global prevalence of mild cognitive impairment among older adults living in nursing homes

Grouping	θ=# studies	df	Q	*χ* ^2^_1_ *_df_*	p-value	*S* ^2^	I^2^
Europe and Central Asia	29	28	0.35	4,682.81	<0.001^1^	4,682.81	-79.00
Upper middle income	8	7	0.75	91.12	<0.001^1^	91.12	-8.33
CAREDiag	2	1	16.51	0.81	0.366^2^	0.81	0.93
Age 70-74	2	1	2.84	0.44	0.505^2^	0.44	0.65
Before year 2000	4	3	4.85	1.23	0.266^2^	1.23	0.38

df, degrees of freedom; CAREDiag, Care Dementia Diagnostic Scale.

1 Refer to heterogenous.

2 Refer to homogeneous.

**Table 2.** Data on parental consent for children receiving vaccination (including Q)

Grouping	θ=# studies	df	Q	*χ* ^2^_1_ *_df_*	p-value^1^	*S* ^2^	I^2^
Lower risk of reporting bias	40	39	0.9976	1,400.78	<0.001	1,117.36	-38.09
High risk of reporting bias	58	57	0.9994	2,877.08	<0.001	2,310.81	-56.03
Developed countries	53	52	0.9993	2,412.97	<0.001	1,935.19	-51.04
Developed countries	45	44	0.9987	1,756.57	<0.001	1,404.57	-43.06
Low income	4	3	0.9961	24.73	<0.001	20.20	-2.01
Upper middle income	29	28	0.9980	753.46	<0.001	596.24	-27.06
High income	62	61	0.9975	3,290.53	<0.001	2,645.56	-60.15
Americas	27	26	0.9982	657.02	<0.001	518.86	-25.05
Southeast Asia	5	4	0.9922	33.33	<0.001	25.40	-3.03
Europe	24	23	0.9949	528.49	<0.001	415.83	-22.12
East Mediterranean	17	16	0.9970	275.30	<0.001	213.93	-15.05
West Pacific	23	22	0.9995	483.19	<0.001	379.66	-21.01
Random sampling	15	14	0.9974	218.20	<0.001	168.69	-13.04
Non-random sampling	74	73	0.9992	4,640.30	<0.001	3,739.76	-72.06
Online data collection	72	71	0.9993	4,396.27	<0.001	3,541.85	-70.05
Self-administered data	13	12	0.9952	168.59	<0.001	129.53	-11.06
Phone interview data	4	3	0.9942	24.82	<0.001	20.28	-2.02
Face to face interview data	9	8	0.9985	86.83	<0.001	65.67	-7.01

df, degrees of freedom.

1 Refer to heterogenous.

From Alimoradi Z, et al. Vaccines (Basel) 2023;11:533 [4].

## Supplementary materials

Supplementary Material 1. A Derivation of the probability structures of the Higgins statistic $I^{2}=\frac{\left(Q-df\right)}{Q}$

Supplementary Material 2. A Proof of *Corr(Q,Y)*=0.

Supplementary Material 3. Critical value of *I*^2^

## References

[1] Nicoll R, Henein MY (2022). COVID-19 prevention: vitamin D is still a valid remedy. J Clin Med.

[2] Glass GV, McGaw B, Smith ML (1981). Meta-analysis in social research.

[3] AlKhafaji D, Al Argan R, Albaker W, Al Elq A, Al-Hariri M, AlSaid A (2022). The impact of vitamin D level on the severity and outcome of hospitalized patients with COVID-19 disease. Int J Gen Med.

[4] Alimoradi Z, Lin CY, Pakpour AH (2023). Worldwide estimation of parental acceptance of COVID-19 vaccine for their children: a systematic review and meta-analysis. Vaccines (Basel).

[5] Alzahrani MA, Almalki F, Aljohani A, Alharbi B, Alsulami B, Alhaddad A (2022). The association between vitamin D serum level and COVID-19 patients’ outcomes in a tertiary center in Saudi Arabia: a retrospective cohort study. Cureus.

[6] Bagiu IC, Scurtu IL, Horhat DI, Mot IC, Horhat RM, Bagiu RV (2022). COVID-19 inflammatory markers and vitamin D relationship in pediatric patients. Life (Basel).

[7] Bahat G, Erbas Sacar D, Petrovic M (2023). Vitamin D in patients with COVID-19: is there a room for it?. Acta Clin Belg.

[8] Borenstein M, Higgins JP, Hedges LV, Rothstein HR (2017). Basics of meta-analysis: I^2^ is not an absolute measure of heterogeneity. Res Synth Methods.

[9] Dantas Damascena A, Galvão Azevedo LM, de Almeida Oliveira T, da Mota Santana J, Pereira M (2023). Vitamin D deficiency aggravates COVID-19: discussion of the evidence. Crit Rev Food Sci Nutr.

[10] Durmuş ME, Kara Ö, Kara M, Kaya TC, Şener FE, Durmuş M (2023). The relationship between vitamin D deficiency and mortality in older adults before and during COVID-19 pandemic. Heart Lung.

[11] Fairfield KM, Murray KA, Anzalone AJ, Beasley W, Khodaverdi M, Hodder SL (2022). Association of vitamin D prescribing and clinical outcomes in adults hospitalized with COVID-19. Nutrients.

[12] Fatemi A, Ardehali SH, Eslamian G, Noormohammadi M, Malek S (2021). Association of vitamin D deficiency with COVID-19 severity and mortality in Iranian people: a prospective observational study. Acute Crit Care.

[13] Grove A, Osokogu O, Al-Khudairy L, Mehrabian A, Zanganeh M, Brown A (2021). Association between vitamin D supplementation or serum vitamin D level and susceptibility to SARS-CoV-2 infection or COVID-19 including clinical course, morbidity and mortality outcomes? A systematic review. BMJ Open.

[14] Hong JY, Kim YJ, Bae S, Kim MK (2023). Associations of daily diet-related greenhouse gas emissions with the incidence and mortality of chronic diseases: a systematic review and meta-analysis of epidemiological studies. Epidemiol Health.

[15] Jenei T, Jenei S, Tamás LT, Putics Á, Knausz M, Hegedüs I (2022). COVID-19 mortality is associated with low vitamin D levels in patients with risk factors and/or advanced age. Clin Nutr ESPEN.

[16] Khan S, Gail M, Samet JM (2020). Statistics for biology and health.

[17] Kolgelier S, Demir NA, Ural O, Sumer S, Kiratli HE, Kirik SY (2022). Vitamin D levels in COVID-19. Acta Medica Mediterr.

[18] Lin LY, Mulick A, Mathur R, Smeeth L, Warren-Gash C, Langan SM (2022). The association between vitamin D status and COVID-19 in England: a cohort study using UK Biobank. PLoS One.

[19] Liu N, Sun J, Wang X, Zhang T, Zhao M, Li H (2021). Low vitamin D status is associated with coronavirus disease 2019 outcomes: a systematic review and meta-analysis. Int J Infect Dis.

[20] Lohia P, Nguyen P, Patel N, Kapur S (2021). Exploring the link between vitamin D and clinical outcomes in COVID-19. Am J Physiol Endocrinol Metab.

[21] Neves FF, Pott-Junior H, de Sousa Santos S, Cominetti MR, de Melo Freire CC, da Cunha AF (2022). Vitamin D deficiency predicts 30-day hospital mortality of adults with COVID-19. Clin Nutr ESPEN.

[22] Bignardi PR, de Andrade Castello P, de Matos Aquino B, Delfino VD (2023). Is the vitamin D status of patients with COVID-19 associated with reduced mortality? A systematic review and meta-analysis. Arch Endocrinol Metab.

[23] Zidrou C, Vasiliadis AV, Tsatlidou M, Sentona M, Vogiatzis S, Beletsiotis A (2022). The relationship between vitamin D status and the clinical severity of COVID-19 infection: a retrospective single-center analysis. Cureus.

[24] Pearson K (1904). Report on certain enteric fever inoculation statistics. Br Med J.

[25] Pérez-Gilaberte JB, Martín-Iranzo N, Aguilera J, Almenara-Blasco M, de Gálvez MV, Gilaberte Y (2023). Correlation between UV index, temperature and humidity with respect to incidence and severity of COVID 19 in Spain. Int J Environ Res Public Health.

[26] Ramirez-Sandoval JC, Castillos-Ávalos VJ, Paz-Cortés A, Santillan-Ceron A, Hernandez-Jimenez S, Mehta R (2022). Very low vitamin D levels are a strong independent predictor of mortality in hospitalized patients with severe COVID-19. Arch Med Res.

[27] Kersh L, Geary K, Roberts M, Daghigh F (2022). Does vitamin D deficiency contribute to COVID-19 severity?. Curr Dev Nutr.

[28] Saygili ES, Karakiliç E (2021). Vitamin D levels and in-hospital mortality of COVID-19. J Health Sci Med.

[29] Purcell N, Sells J, McGrath S, Mehlman H, Bertenthal D, Seal KH (2021). “Then COVID happened…”: veterans’ health, wellbeing, and engagement in whole health care during the COVID-19 pandemic. Glob Adv Health Med.

[30] Thacher TD (2022). Evaluating the evidence in clinical studies of vitamin D in COVID-19. Nutrients.

[31] Walczak K, Walczak P, Zdun S, Nemeczek S, Merkisz K, Grzybowski J (2023). Effect of vitamin D on the course of COVID-19 infection. J Educ Health Sport.

[32] Chernikova O, Heitzmann N, Stadler M, Holzberger D, Seidel T, Fischer F (2020). Simulation-based learning in higher education: a meta-analysis. Rev Educ Res.

[33] Blumenfeld D (2009). Operations research calculations handbook.

[34] Chen P, Cai H, Bai W, Su Z, Tang YL, Ungvari GS (2023). Global prevalence of mild cognitive impairment among older adults living in nursing homes: a meta-analysis and systematic review of epidemiological surveys. Transl Psychiatry.

[35] Chen J, Mei K, Xie L, Yuan P, Ma J, Yu P (2021). Low vitamin D levels do not aggravate COVID-19 risk or death, and vitamin D supplementation does not improve outcomes in hospitalized patients with COVID-19: a meta-analysis and GRADE assessment of cohort studies and RCTs. Nutr J.

[36] Higgins JP, Thompson SG, Deeks JJ, Altman DG (2003). Measuring inconsistency in meta-analyses. BMJ.

