# Supplementary material for: The bounds of meta-analytics and an alternative method
Source: Epidemiol Health. 2024 Jan 7;46:e2024016. doi: 10.4178/epih.e2024016 (PMC11040225; doi:10.4178/epih.e2024016)
Supplement: Supplementary Material 1. — A Derivation of the probability structures of the Higgins statistic I2=Q-dfQ [file epih-46-e2024016-Supplementary-1.docx]

**The bounds of meta-analytics and an alternative method**

**Supplementary Materials: S1, S2, S3**

**S1. A Derivation of the probability structures of the Higgins statistic** $I^{2}=\frac{(Q - df)}{Q}$

In this section, we first derive the probability density function of $I^{2}$and then explore its properties for discussion relevant to the main theme of this article. Let the random number, $Y=y$denote the number of studies on a meta topic available for consideration at a point of time. Realize that the number of studies grows unboundedly in any time depending on the research curiosity of professionals in the topic. Notice that the observable space for the number of studies in meta-analysis is $y=2,3,...,\theta$, where $\theta$ is an unknown upper-bound parameter. That is, the uniform probability mass function, $\Pr(Y=y\left|\theta\right.)$for the random number of studies to be included in a meta-analysis is $\Pr(Y=y\left|\theta\right.)=(\theta-1)^{-1};y=2,3,...,\theta$so that the total probability is $\sum_{y=2}^{\theta} \Pr(Y=y\left|\theta\right.)=(\theta-1)^{-1}\sum_{y=2}^{\theta} 1=(\theta-1)^{-1}[\{\sum_{y=1}^{\theta} 1\}-1]=(\theta-1)^{-1}(\theta-1)=1$.

By nonnegative variable transformation $u=\frac{y-1}{\theta}$, we notice that the probability pattern of the transformed variable, $u$is a lower-truncated continuous uniform with the sample space $\frac{1}{\theta}<u<1$and probability density function (pdf) $f(u\left|\theta\right.)=(1-\frac{1}{\theta})^{-1}$satisfying the requirements that $f(u\left|\theta\right.)$is non negative and $\int_{\frac{1}{\theta}}^{1} f(u\left|\theta\right.)du$=1.

By applying an additional transformation $w=-2\ln u$, we note that not only the observable space changes to $0<w<-2\ln(\frac{1}{\theta})$ but also the pdf changes to a conjugate $f(w\left|\theta\right.)=(1-\frac{1}{\theta})^{-1}(\frac{1}{2})e^{-(\frac{w}{2})}$satisfying the requirements that$f(w\left|\theta\right.)$is non-negative and$\int_{0}^{-2\ln(\frac{1}{\theta})} f(w\left|\theta\right.)dw=1$. Figure 1 is the sketch of the pdf $f(w\left|\theta\right.)$.


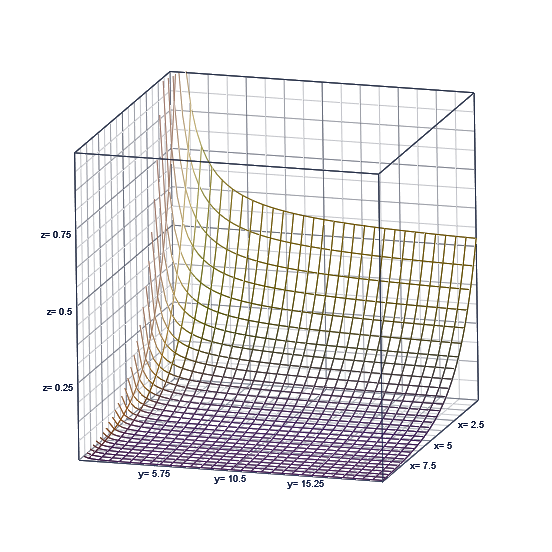


**Figure 1. The pdf $f(w\left|\theta\right.)$with $w$ in x-axis and $\theta$ in y-axis.**

Now, we derive the expected value of the transformed random variable, $W$. We note that

$E(w\left|\theta\right.)=\int_{0}^{-2\ln(\frac{1}{\theta})} wf(w\left|\theta\right.)dw=(1-\frac{1}{\theta})^{-1}(\frac{1}{2})\int_{0}^{-2\ln(\frac{1}{\theta})} we^{-(\frac{w}{2})}dw$.

The integral in the above expression is an incomplete gamma function. To find a finite (approximate) result for the integral, we write $\ln(\frac{1}{\theta})=\ln(1-[1-\frac{1}{\theta}])\approx-(1-\frac{1}{\theta})$ due to the Taylor’s series as $\frac{1}{\theta}<1$ because$\theta\geq1$. Notice that the expectation simplifies to $E(w\left|\theta\right.)\approx(1-\frac{1}{\theta})$, which is sketched in Figure 2.


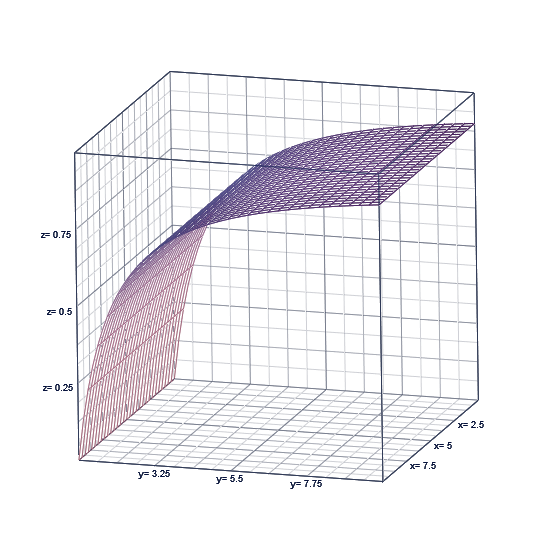


**Figure 2. $E(w\left|\theta\right.)$in z-axis with $\theta$in y-axis.**

Now, we proceed to find a finite (approximate) result. By continuation of the same approximation again as seen above, we find that$E(w^{2}\left|\theta\right.)\approx4(1-\frac{1}{\theta})^{2}$. Substituting the above results in the variance$Var(w\left|\theta\right.)=E(w^{2}\left|\theta\right.)-\{E(w\left|\theta\right.)\}^{2}$and simplifying, we obtain that$Var(w\left|\theta\right.)\approx3\{E(w\left|\theta\right.)\}^{2}$which is sketched in Figure 3.


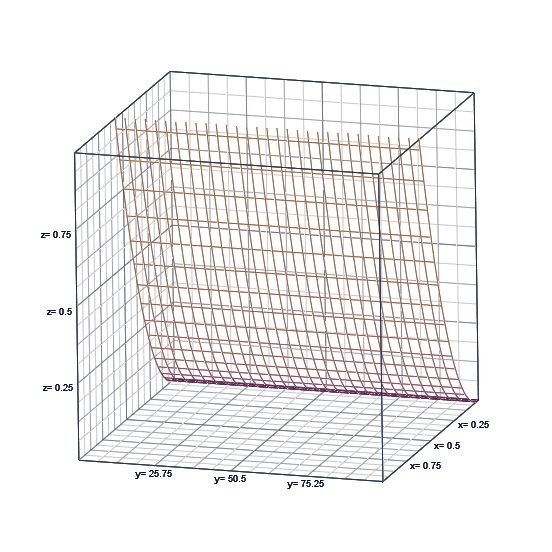


**Figure 3. z = $Var(w\left|\theta\right.)\approx3\{E(w\left|\theta\right.)\}^{2}$with $E(w\left|\theta\right.)$in x-axis**

We now proceed to understand the survival function of$w$and it is $\Pr[W>m]=1-(1-\frac{1}{\theta})^{-1}(1-e^{-m/2});m>0$ which is sketched in Figure 4 below.


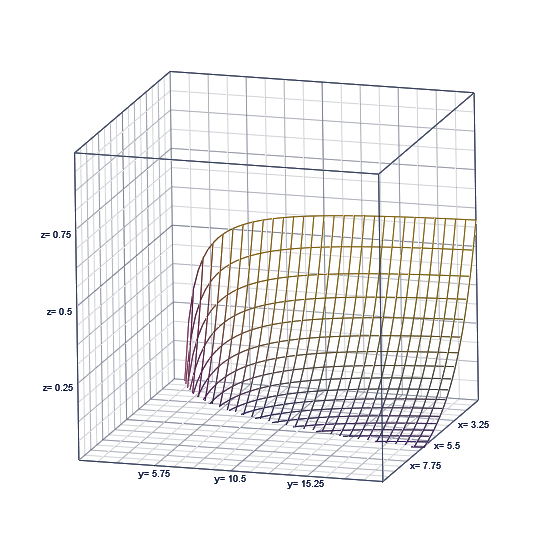


**Figure 4. The survival function $\Pr[W>m]$ with $m$in x-axis and $\theta$ in y-axis**

The incremental rate of doing additional studies by the researchers is

$$h[w\left|m\right.]=\frac{f(w\left|\theta\right.) }{\Pr[W>m]}=\frac{e^{-(\frac{w}{2})}}{2[\{1-\frac{1}{\theta}\}-\{1-e^{-(\frac{w}{2})}\}]}$$

which stabilizes at the asymptote $[1+e^{(\frac{m}{2})}(1-\frac{2}{\theta})]^{-1}$when w catches up to $m\geq2$. We shall now focus on the conditional probability pattern in the data oriented statistics $Q$ for a given $w$, where $w$is another version of $y$. The reparameterizations proceed in steps as follows. The number of studies, y ranges from two to $\theta$. Note that $u=\frac{y}{\theta}$ ranges from $\frac{1}{\theta}$ to one. With $w=-2\ln u$ ranges from zero to $2\ln\theta$. We now cast these in terms of our notations. That is, from these transformations, we notice that the degrees of freedom equal to $df=y-1=\{\theta e^{-(\frac{w}{2})}-1\}$. The probability density function (pdf) of $Q$ in terms of a reparametrized version $0<w<2\ln\theta$ is.

$f(Q\left|w\right.)=e^{-(\frac{Q}{2})}Q^{(\frac{\{\theta e^{-(\frac{w}{2})}-1\}}{2})-1}/2^{(\frac{\{\theta e^{-(\frac{w}{2})}-1\}}{2})}\Gamma(\frac{\{\theta e^{-(\frac{w}{2})}-1\}}{2});Q>0;0<w<2\ln\theta;\theta\geq1$ (1)

It is a simple algebraic exercise to prove the requirements,$f(Q\left|w\right.)>0$and$\int_{0}^{2\ln\theta} f(Q\left|w\right.)dw=1$.

Note that $E(Q\left|w\right.)=\{\theta e^{-(\frac{w}{2})}-1\}$for the sketch of the pdf $f(w\left|\theta\right.)$. $E(Q\left|w\right.)=\{\theta e^{-(\frac{w}{2})}-1\}$with $\theta$ in the y-axis and $w$in x-axis in Figure 5.


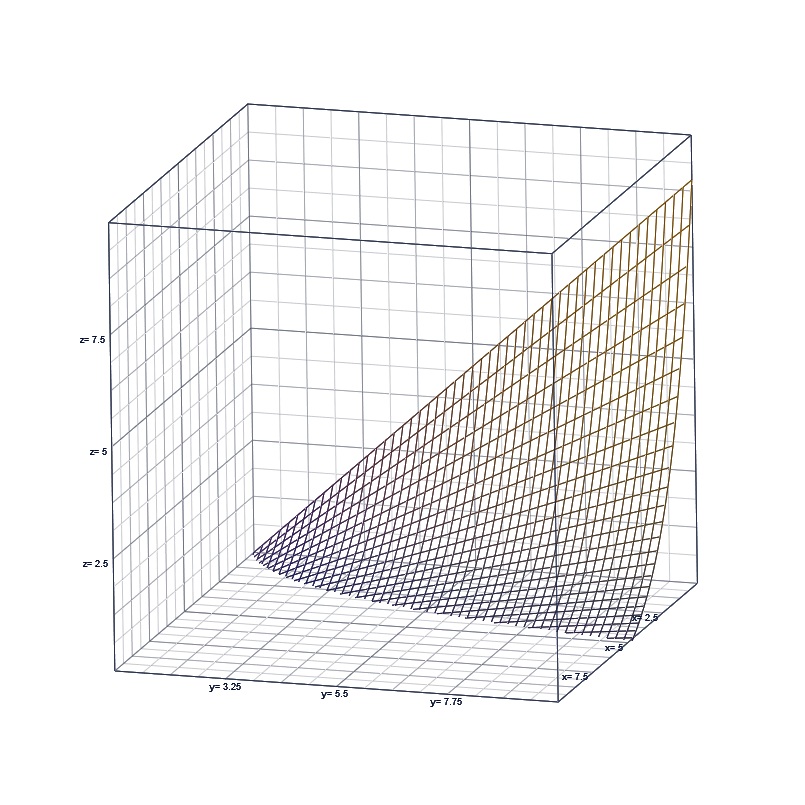


**Figure 5. Note that $Var(Q\left|w\right.)=2E(Q\left|w\right.)$.**

Using the known results $E(Q)=E_{w}E(Q\left|w)\right.$ and $Var(Q)=E_{w}Var(Q\left|w)\right.+Var_{w}E(Q\left|w)\right.$, the unconditional expected value and variance of the statistic, $Q$, are, respectively,

$E(Q)=E_{W}E(Q\left|w\right.)=E_{W}\{\theta e^{-(\frac{w}{2})}-1\}=(\frac{\theta-1}{2})$ (2)

and

$Var(Q)=E_{w}Var(Q\left|w)\right.+Var_{w}E(Q\left|w)\right.=\frac{(\theta+11)(\theta-1)}{12}.$ (3)
