# Supplementary material for: The bounds of meta-analytics and an alternative method
Source: Epidemiol Health. 2024 Jan 7;46:e2024016. doi: 10.4178/epih.e2024016 (PMC11040225; doi:10.4178/epih.e2024016)
Supplement: Supplementary Material 2. — A Proof of Corr(Q,Y)=0. [file epih-46-e2024016-Supplementary-2.docx]

**S2. A Proof of $Corr(Q,Y)=0$.**

In this section, we prove that the statistics Q and Y are not correlated. To prove it, we proceed as follows. That is,$Cov(df,Q)=E_{w}E_{Q\left|w\right.}\{(\theta e^{-(\frac{w}{2})}-1)Q\left|w\right.\}-E_{w}(\theta e^{-(\frac{w}{2})}-1)E(Q)$ with$df=y-1=\{\theta e^{-(\frac{w}{2})}-1\}$. For this result, note that $E_{Q\left|w\right.}\{(\theta e^{-(\frac{w}{2})}-1)Q\left|w\right.\}=(\theta e^{-(\frac{w}{2})}-1)^{2}$due to the fact that$E(Q\left|w\right.)=\{\theta e^{-(\frac{w}{2})}-1\}$, $E(e^{-w})=\frac{1}{3}(1+\frac{1}{\theta}+\frac{1}{\theta^{2}})$, and $E(e^{-(\frac{w}{2})})=\frac{1}{2}(1+\frac{1}{\theta})$. Consequently, $E_{w}E_{Q\left|w\right.}\{(\theta e^{-(\frac{w}{2})}-1)Q\left|w\right.\}=E_{w}(\theta e^{-(\frac{w}{2})}-1)^{2}=\frac{(\theta-1)^{2}}{3}$. In other words, $E(Q)=E(df).$ Hence, $Cov(df,Q)=0$ and $Corr(df,Q)=0$.
