# Supplementary material for: The bounds of meta-analytics and an alternative method
Source: Epidemiol Health. 2024 Jan 7;46:e2024016. doi: 10.4178/epih.e2024016 (PMC11040225; doi:10.4178/epih.e2024016)
Supplement: Supplementary Material 3. — Critical value of I2 [file epih-46-e2024016-Supplementary-3.docx]

**S3. Critical value of** $I^{2}$

To obtain the critical value of $I^{2}$, we utilize an expression, from Blumenfeld [1], page 8. That is, when two random variables U and V are independent, $E(\frac{U}{V})=[\frac{E(U)}{E(V)})(1+\frac{Var(V )}{[E(V)]^{2}})$ and $Var(\frac{U}{V})=[\frac{E(U)}{E(V)}]^{2}[\frac{Var(U)}{[E(U)]^{2}}+\frac{Var(V )}{[E(V)]^{2}})$. Letting $U=df$and$V=Q$ in Higgins $I^{2}$score, we note that$E(I^{2})=1+(1-\frac{Var(Q)}{[E(Q)]^{2}})=2-\frac{(\theta+11)}{6(\theta-1)}$ and

$$Var(I^{2})=Var(\frac{df}{Q})=\frac{Var(df)}{[E(df)]^{2}}+\frac{Var(Q)}{[E(Q)]^{2}}=\frac{1}{3}[1-\frac{(\theta+11)}{2(\theta-1)}]$$

because$E(df)=\frac{(\theta-1)}{2}=E(Q)$. This means that a new statistic,

$S^{2}=[\frac{I^{2}-E(I^{2})}{\sqrt{Var(I^{2})}}]^{2}=\chi_{1df}^{2}=[\frac{\{\frac{(\theta+11)}{6(\theta-1)}-(1+\frac{df}{Q})\}}{\sqrt{3\left|1-\frac{(\theta+1)}{2(\theta-1)}\right|}}]^{2}$ (4)

could be utilized as it follows a chi-squared distribution with one degrees of freedom (df). In other words, the $p-value$of a data base $S^{2}$is $\Pr(S^{2}\geq\chi_{1,p-value}^{2})=p-value$. These results would help the practitioner to have more confidence in conducting meta-analysis.

REFERENCES

1. Blumenfeld D. Operations research calculations handbook. Boca Raton: CRC Press; 2009, p. 8-9.
